# Changing friction at the base of an Alpine glacier

**DOI:** 10.1038/s41598-021-90176-9

**Published:** 2021-05-25

**Authors:** Dominik Gräff, Fabian Walter

**Affiliations:** Laboratory of Hydraulics, Hydrology and Glaciology (VAW), ETH Zürich, Zürich, Switzerland

**Keywords:** Seismology, Cryospheric science

## Abstract

Repeating earthquakes are a global phenomenon of tectonic faults. Multiple ruptures on the same fault asperities lead to nearly identical waveforms characteristic for these seismic events. We identify their microseismic counterparts beneath an Alpine glacier, where basal sliding accounts for a significant amount of ice flow. In contrast to tectonic faults, Alpine glacier beds are subject to large variations in sliding velocity and effective normal stresses. This leads to inter- and sub-seasonal variations in released seismic moment from stick–slip asperities, which we explain with the rate-and-state friction formalism. During summer, numerically modelled effective normal stresses at asperities are three times higher than in winter, which increases the local shear resistance by the same factor. Stronger summer asperities therefore tend to form in bed regions well connected to the efficient subglacial drainage system. Moreover, asperities organise themselves into a state of subcriticality, transferring stresses between each other. We argue that this seismic stick–slip behavior has potentially far-reaching consequences for glacier sliding and in particular for catastrophic failure of unstable ice masses.

## Introduction

Processes controlling glacier and ice stream dynamics occur at or close to the ice-bed interface^1^. These basal processes regulate stagnation or acceleration of polar ice streams^2,3^, are responsible for seasonal speed up of tidewater outlet glaciers^4^, initialize local instabilities and enable their propagation as surges along the entire glacier bed^5^. This may cause catastrophic failure of basal resistance resulting in break-off events^6^ or runaway surges during which entire glacier tongues detach from their beds^7^.

In conventional glacier sliding theories, normal traction at perfectly lubricated bed obstacles controls basal sliding by enhanced viscous creep and melt-refreeze cycles^8^. On soft beds, till deformation and true basal sliding of the ice over the till surface takes place^9,10^. Though initially ignored in sliding investigations, dynamically changing friction at the bed may also play a critical role in basal sliding: Sudden sliding events of Antarctic ice streams suggest a frictional resistance, which evolves over sub hourly time scales^11^. Laboratory experiments confirm that under certain conditions, rate-weakening friction at the ice stream bed can explain sliding instabilities and thus the episodic ice stream acceleration^12,13^. Moreover, sliding episodes seem to be the sum of countless microseismic stick–slip events, which themselves are a manifestation of rate-weakening friction^14^.

In recent years, microseismic stick–slip events have been observed at fast ice streams^15–17^ as well as slowly moving parts of the Greenland ice sheet and Alpine glaciers^18–20^. Microseismic stick–slip events tend to cluster at distinct bed locations producing nearly identical seismic waveforms and repeated ruptures may coalesce into sustained tremor-like signals^16,21^. The clustering behavior can be explained with rate-weakening friction asperities embedded within an otherwise smoothly sliding, rate-strengthening ice-bed interface, a concept, which has been extensively studied in the context of tectonic faults^22^.

The underlying theoretical principles are still debated but have to include a dependence on subglacial water pressures supported by decades of observations^23,24^. In this regard, the configuration of the subglacial drainage system is of primary importance, as it can consist of efficient channels operating under low pressures or of networks of smaller, inefficient and pressurized drainage pathways^25^. Depending on water availability, subglacial pressures and drainage channels evolve over seasonal or sub seasonal time scales: water-filled cavities, which are part of the inefficient drainage system, open during periods of high subglacial water pressure and stay open throughout the melt season reducing the contact forces between ice and bed thus enhancing sliding^26^. In contrast, efficient drainage channels characteristic for the melt season are more dynamic and react to diurnal variations in meltwater supply from the surface^27^. The spatially and temporarily varying hydraulic regimes have to be accounted for in realistic theories of basal sliding. Although microseismic stick–slip events seem to react to meltwater input^20^, a systematic analysis of stick–slip activity is still needed to clarify if dynamic friction satisfies the hydraulic constraints on basal sliding.

In this study, we investigate the interplay between frictional glacier sliding and varying sub-glacial conditions beneath an Alpine glacier. Stick–slip asperities react to seasonal changes in glacial melt water supply and organize themselves into a sub-critical state in which they react to each other’s activity over hundreds of meters. Elucidating how stresses and sliding velocity at the glacier bed change on inter- and sub-seasonal time scales, we show that rate-and-state friction developed for tectonic earthquake cycles is a viable theoretical framework to describe basal sliding.

## Results

### Study site and data acquisition

**Figure 1:**
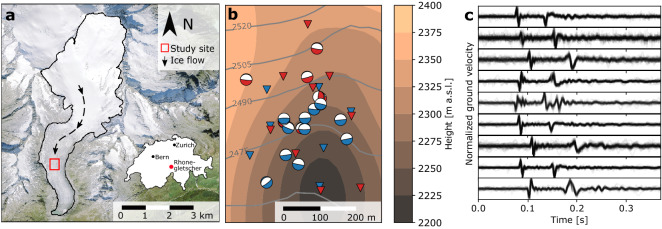
Study site with stick–slip asperities and seismogram. (**a**) Orthoimage of Rhonegletscher and study site. (**b**) Zoom into the red rectangle of a. Background shows the bedrock topography from interpolated ground-penetrating-radar surveys^29^. Surface topography is indicated by gray contours. Red (blue) triangles indicate seismic sensor array in summer (winter). Red (blue) beach balls show focal mechanisms of individual summer (winter) stick–slip clusters. Zoom into dense asperity region is shown in Supplementary Fig. S6. (**c**) Vertical seismograms of events from one cluster. Source of orthoimage in a: Swiss Federal Office of Topography.

Our study site is located in the ablation zone of Rhonegletscher (Switzerland) at an elevation of ~ 2500 m above sea level, where the glacier is ~ 200 m thick and at the pressure melting point (rectangle in Fig. 1a at WGS84: 46.597, 8.382)^28^. As shown below, seasonal and diurnal variations in surface velocity indicate a significant contribution of basal sliding to ice flow. During a winter measurement period (February 13th–March 20th, 2018) and a summer period (July 21st–August 22nd, 2018), we deployed seismometer arrays consisting of seven and nine seismometers, respectively, forming 300–500 m large apertures (Fig. 1b and Supplementary Table S1). Three stations were co-located in both years and during summer were equipped with GPS antennae for measurements of surface displacements.

### Basal microseismic stick–slip asperities

During both measurement periods, we recorded basal microseismic stick–slip events (M_w_ ≈ −3 to −2, i.e. very weak) with shear faulting source mechanisms indicated by mixed P-wave polarities throughout the arrays. We detect the stick–slip signals with a spectral discriminator, apply hierarchical clustering to identify events with identical waveforms and perform a template match search on the continuous data (Methods/Supplementary Fig. S1, S2, S3, S4, S5). Using a probabilistic nonlinear hypocenter location scheme^30^ we locate stick–slip sources accounting for picking and velocity model uncertainties (Supplementary Table S4).

The 1158 stick–slip events recorded in summer form seven clusters, the 2025 winter events form eleven clusters (Fig. 1b), with each cluster containing events of nearly identical waveforms (Fig. 1c). Each cluster is associated with the hypocentral location of an asperity, where bed properties allow for the accumulation of elastic strain, which is released during stick–slip events^12^. The summer and winter cluster locations are not identical, showing that permanent topographic bed features are not the primary control of stick–slip events, in contrast to ice shelf pinning points in Antarctica producing stick–slip events^31^. Location uncertainties of three asperities from winter and summer overlap, however comparison of waveforms shows that locations differ slightly (Supplementary Fig. S6). This indicates that on small spatial scales, subglacial conditions favoring stick–slip asperities change between the seasons.

On average, the seismic moment rate ($\mathop {{\textit{M}}_{0} }\limits^{ \cdot }$) released from summer asperities is three times higher compared to winter asperities. Seismic moments of events from winter asperities are typically half a magnitude lower than during summer. However, melt-induced seismic background noise reduces detectability of low-magnitude stick–slip events in summer^32^. Thus, undetected events with weak seismic moments comparable to winter events may exist during summer. This means that our calculated seasonal difference in $\mathop {{\textit{M}}_{0} }\limits^{ \cdot }$ is a lower bound.

### Stick–slip event scaling relation

**Figure 2:**
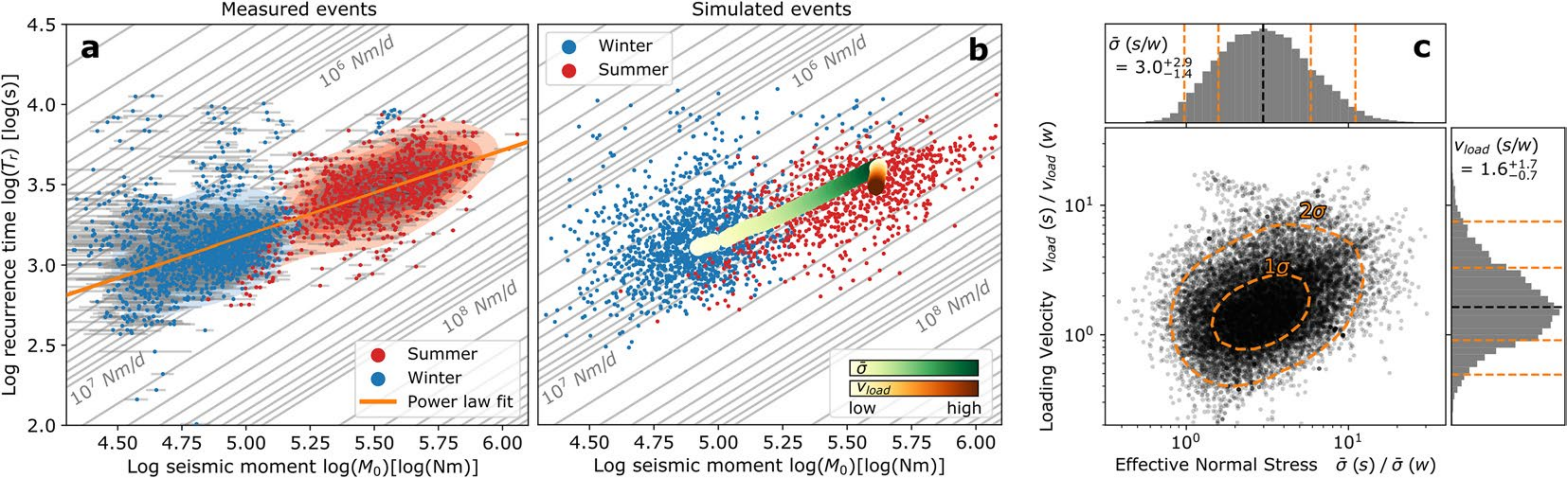
Recurrence time-seismic moment scaling relation. (**a**) Double-logarithmic scatter plot of measured intra-asperity recurrence time versus event seismic moment for summer (red) and winter (blue) events. Orange line indicates the $1\sigma$ area of a power law fit. Along gray diagonal lines, recurrence times and seismic moment change while seismic moment rate is constant $\mathop { M_{0} }\limits^{ \cdot } = M_{0} /T_{r} = const.$→ $log\left( {T_{r} } \right) = log\left( {M_{0} } \right) - \log \left( {\mathop {M_{0} }\limits^{ \cdot } } \right) + log\left( {86400} \right)$. Plotted moment rates follow a logarithmic scale increasing towards the lower right and selected values are labeled. (**b**) Same as a, but with simulated data points. Green and red paths describe a threefold increase in effective normal stress and 1.6-fold increase in loading velocity that is needed to describe recurrence time and seismic moment increases from winter to summer. (**c**) Double-logarithmic scatter plot showing the parameters that were adjusted to reproduce the data in a. The x-axis shows the ratio of effective normal stress in summer vs. winter, the y-axis shows the ratio of the loading velocity in summer vs. winter. Histograms show the one-dimensional distribution of the scatter points. Orange dashed lines indicate $1\sigma$ and $2\sigma$ uncertainty regions.

Within individual asperities, winter events are weaker with shorter recurrence time compared to summer. Combined, they follow a scaling relation between recurrence time $T_{r}$ and seismic moment $M_{0}$ of $T_{r} \propto M_{0}^{0.53 \pm 0.01}$ implying that the rate of seismic moment release is higher in summer compared to the winter season (Fig. 2a).

The measured dependence of event recurrence on seismic moment is approximately three times as strong as what is expected for a seismogenic strike-slip fault that also slips aseismically, which is $T_{r} \propto M_{0}^{1/6}$^22^. It is still 1.5 times stronger than for a purely seismically slipping fault for which one would expect $T_{r} \propto M_{0}^{1/3}$^33^. However, the comparison to scaling relations for tectonic faults is questionable, because in our alpine glacier setting, environmental parameters controlling both recurrence time and seismic moment vary substantially more. Seasonal and daily sliding velocities, which are responsible for elastic loading on basal asperities, vary by up to 100%^34^. Most importantly, depending on surface melt production, subglacial water pressures may vary by one hundred meter of water column over the course of a few hours and locally reach flotation level during peak pressures^35^. As a result, the seismic stress drop at stick–slip asperities is not constant such that a simple normalization by the sliding velocity^36^ cannot explain the observed scaling between $T_{r}$ and $M_{0}$ and would result in even higher scaling exponents (Supplementary Notes). Therefore, changing conditions at the glacier bed including changing effective normal stresses have to be considered.

### Modelling summer–winter scaling

On frictional faults, sliding and cycles of quiescence and seismic activity are described by rate-and-state laws relating the coefficient of friction $\mu$ to an evolving interface state and the sliding velocity^37^. Seismogenic sliding arises as an instability when friction decreases with increasing sliding velocity (‘rate-weakening’). In contrast, rate-strengthening friction tends to stabilize sliding via increased frictional drag and therefore arrests transient increases in sliding velocity.

Rate-and-state friction is commonly described by the logarithmic law

1 $$ \mu = \mu_{0} + a\;ln\left( {v/v_{0} } \right) + b\;ln\left( {v_{0} \theta /L} \right) $$

where $v$ is the sliding velocity, $v_{0}$ is the steady-state sliding velocity such that $\mu = \mu_{0}$ when $v = v_{0}$. *a, b* are material parameters of the interface and rate-weakening holds when $a < b$. The critical slip distance *L* is often interpreted as a memory distance over which the population of small-scale contacts between the two fault sides changes^38,39^. ${\theta}$ is the state variable that represents an average contact lifetime and which is described by an evolution law^37^:

2 $$ \frac{d\theta }{{dt}} = 1 - \frac{\theta v}{L} $$

We use (1) in combination with (2) in order to model our observed scaling of recurrence time and seismic moment of stick–slip events and in particular the difference between summer and winter measurements (Fig. 2a). The effect of varying subglacial water pressures is captured by equating ${\mu }$ to the ratio of effective normal stress $\overline{\sigma }$ (normal stress minus subglacial water pressure) and shear stress $\tau$ at the bed:

3 $$ \tau = \mu \cdot \overline{\sigma } $$

Sliding velocities vary in response to these pressure variations^24,26^. The parameters *L*, *a, b* are difficult to constrain, but laboratory measurements have established conditions resulting in rate-weakening beds for ice at the pressure melting point and specific interface conditions^40^.

The rate-and-state formalism describes friction as velocity dependent and allows interface strengthening (‘healing’) between events. This means that simple failure thresholds for sliding velocity or shear stress do not exist, but that stick–slip initiation depends nonlinearly on various parameters, including the time elapsed since the previous event. Rate-and-state friction is a semi-empirical formulation, which holds for different materials in laboratory and natural faults. Physical interpretation for rate-strengthening and weakening parametrized by *L*, *a* and *b* are found in characteristic material behavior. For ice-bed contacts, healing may be related to pressure-enhanced melting at contacts between ice and small-scale bed bumps^41^. Regelation and viscous ice flow near the ice-bed interface are candidates for rate-strengthening mechanisms^8^, whereas sediment entrainment at the ice sole can lead to rate-weakening^12,42^.

In order to simulate the observed scaling between seismic moment and recurrence time, we vary effective normal stress and loading velocity (rate of build-up shear stress at asperity) between pairs of summer and winter simulations while keeping *L*, $v_{0} ,\;\mu_{0} ,$ *a*, and* b* constant within these pairs (Supplementary Table S3). Our numerical implementation of rate-and-state friction is based on a 1D spring-loaded slider-block, which simulates the stick–slip asperity via the block-bed contact and lumps elastic stresses within the ice and bed into the spring force. This model disregards the influence of fault area, which we expect to be dictated by regular bedrock undulations filled with subglacial till^43^ and thus to remain constant between summer and winter. Moreover, by averaging seismic moments and recurrence times over all asperities, local deviations from mean fault areas or material composition at individual asperities are expected to cancel out. The assumption of a constant fault area leads to a proportionality between the seismic stress drop $\Delta {\tau }_{s}$, and the measured seismic moment $M_{0}$ (Stein and Wysession, 2003):

4 $$ \Delta {\tau }_{s} = \frac{7}{16}\frac{{M_{0} }}{{D^{3} }} \Rightarrow \Delta {\tau}_{s} \propto M_{0} \quad {\text{for fault dimension}}\;D = const. $$

We apply Bayes’ theorem^44^ with data variance given by our spread in moment and recurrence time measurements and priors listed in the Supplementary Table S3. As a result, the effective normal stress at the basal asperities during summer is expected to be a factor of $3.0_{ - 1.4}^{ + 2.9}$ larger than in winter, and summer sliding velocities are required to be $1.6_{ - 0.7}^{ + 1.7}$ times larger than in winter (Fig. 2c). Uncertainties of one standard deviation are given and arise primarily from overlapping summer/winter data points (Fig. 2a, b).

**Figure 3:**
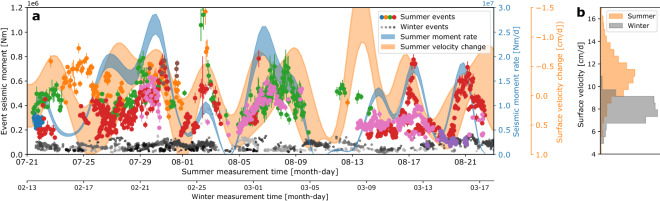
Stick–slip activity time series. (**a**) Time series of stick–slip events for the summer (different color dots) and winter measurement periods (gray dots with time scale on the bottom). Blue area indicates the released seismic moment for the summer events with $1\sigma$ uncertainty. Yellow area indicates the measured surface velocity during the summer measurement period with $1\sigma$ uncertainty. (**b**) Normalized histogram of surface velocity measurements during summer and winter. The difference is attributed to basal sliding.

An increase in basal loading velocity for the summer season agrees with surface velocity measurements (Fig. 3b). No simple measurement exists to confirm the calculated increase in effective normal stress at the asperities. However, the increase shows that during summer, subglacial water pressures at asperities are lower such that effective normal stresses increase. This can be explained by efficient drainage channels operating under low water pressures^27^ and nearby asperities which highlights the hydraulic control on frictional resistance to ice flow.

### Sub-seasonal stick–slip variations

We compare seismic moment release over six-hour windows summed over all asperities to surface velocity variations (Fig. 3a). Both time series were bandpass filtered (Methods) to suppress fluctuations of the diurnal melt cycle and velocity variations thus reflect longer term trends in surface melt water production and the connectivity of un-channelized regions of the bed^45^. Measured surface velocity variations result from sliding variations and mirror released seismic moment rate: Within one or two days, moment rate maxima and minima respectively coincide with minima and maxima in surface velocity (Fig. 3a, note the flipped velocity axis). Surface velocity decreases when the glacier lowers due to falling water pressures. This can be explained by the increase of contact area between the glacier ice and the underlying bed^46^ resulting in increased frictional resistance at asperities.

Higher effective normal stresses at the asperities increase recurrence times. This, in turn, increases fault healing, which overcompensates decreasing loading velocities to induce higher seismic moment rates. This overcompensation is another manifestation of the rate-and-state effect responsible for the winter-to-summer increase in effective pressure, which is around twice as large as the concurrent loading velocity increase ($3.0_{ - 1.4}^{ + 2.9}$ compared to $1.6_{ - 0.7}^{ + 1.7}$).

During episodes of fast sliding and corresponding low seismic moment rates, effective stresses at the asperities reduce due to pressurization of the subglacial discharge system and can locally even lead to uplift of the ice column^23^. Accordingly, effective stresses may fall below a critical value $\overline{{{\sigma }_{{\text{c}}} }}$ which for a spring-loaded slider-block model with spring constant *k* is $\overline{{{\sigma }_{{\text{c}}} }} = {\textit{kL}}/\left( {b - a} \right)$^37^. In this case, even for rate-weakening bed material, frictional sliding proceeds aseismically.

## Discussion

**Figure 4:**
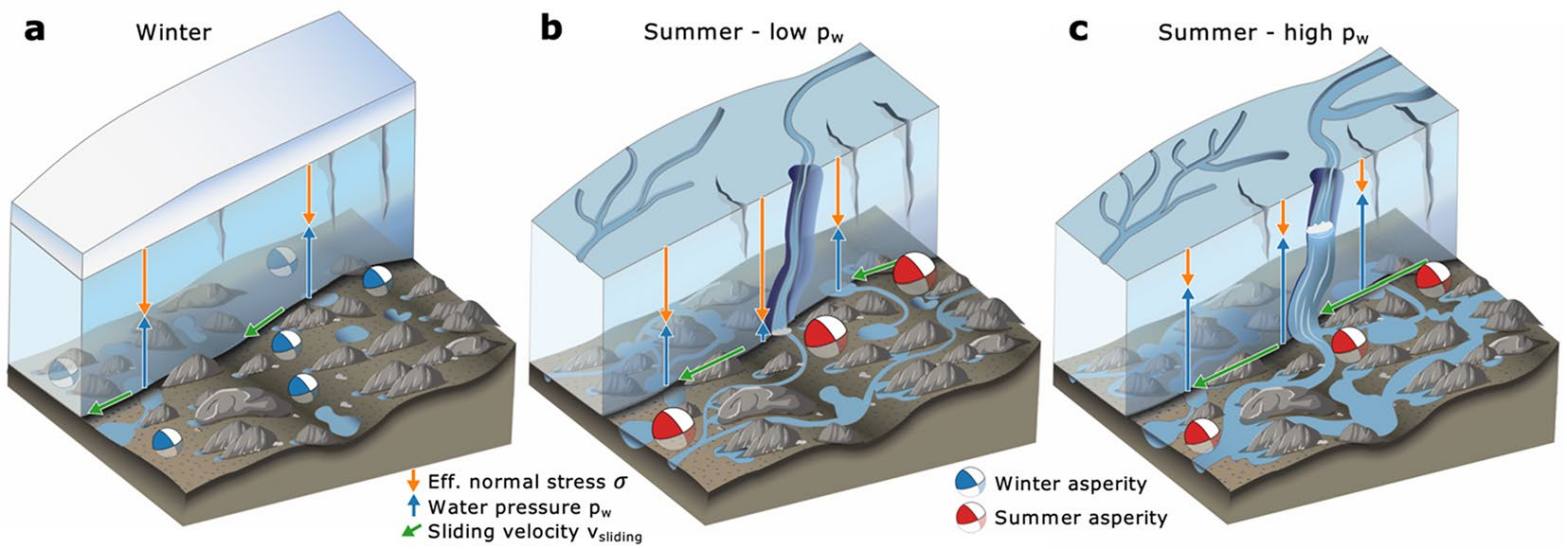
Conceptual model of subglacial hydraulic system and asperities therein. (**a**) Subglacial environment in winter. Isolated cavities dominate the subglacial hydraulic system. Sliding velocity is low and effective normal stresses and stick–slip asperities are evenly distributed over the bed (**b**) Summer conditions during phases with low basal water pressure. Linked cavities have opened up and constitute the non-efficient subglacial drainage system that enables high sliding velocities even during low water pressures in channels. Stick–slip asperities develop close to these channels and release high moments. (**c**) Similar to b, but with high basal water pressures resulting in high sliding velocities and less moment released at stick–slip asperities compared to summer conditions with low water pressures shown in panel b.

Our analysis reveals differences in seismogenic glacier sliding at seasonal and sub-seasonal time scales. Rate-and-state friction explains these observations in terms of variations in glacier loading velocity and effective normal stresses. On seasonal timescales, both parameters act in accord to increase stick–slip magnitudes more strongly than recurrence times resulting in higher moment rates in summer. On the other hand, for multi-day variations in stick–slip activity during summer, loading velocity and effective normal stresses compete, and the latter dominates to increase moment rate during decelerating sliding by the effect of fault healing. In this picture, stick–slip asperities form preferentially at bed regions, which are well connected to efficient subglacial drainage channels and thus are subject to higher effective pressures and variations thereof (Fig. 4)^27^. Basal sliding velocities are driven by pressurized subglacial cavities which dampen the response of sliding to changing water pressures in the efficient drainage system^26,45^. These findings highlight the inhomogeneous hydraulic control on basal resistance to ice flow in the presence of an efficient subglacial drainage system. Our 1D rate-and-state friction model requires a tripling in effective normal stress for summer conditions compared to winter, but only 1.6-fold increase in loading velocities (Fig. 2).

The concept of hydraulically-induced sliding variations is not new^24,26,47^. However, our findings show that high effective normal stresses and hence high shear resistance focus at discrete patches acting as microseismic stick–slip asperities^48^. The effect of this stress concentration on large-scale basal resistance to ice flow depends on whether basal motion at stick–slip asperities is entirely seismic or contains other mechanisms such as enhanced viscous ice deformation of the ice sole. Even for the latter case, these stress concentrations will locally affect basal motion, since ice deformation depends on effective stress: Close to flotation level, our calculated threefold change in effective stress can result in order-of-magnitude changes in effective viscosity of temperate ice^49^.

The presented stick–slip catalogue shows that at least parts of the glacier bed behave like tectonic faults subject to rate-and-state friction ^37^. Glacial data sets therefore provide new observational constraints on numerical rate-and-state friction models, because in contrast to tectonic faults, they are subject to large variations in effective normal stress and loading velocity^36^ resulting in varying static stress drops. The correspondence between moment rate extrema and velocity variations in summer (Fig. 3) indicates that stick–slip asperities influence each other’s behavior over distances of at least 100–200 m: different asperities contribute at different times to moment rate maxima, such that reduced seismic activity of one asperity is compensated by increased activity of another asperity. This constitutes a self-organized system where overall structural order forms without an external control^50^. Stress transfer between asperities occurs on daily or even sub-daily timescales. This is around or above the Maxwell relaxation time, which is on the order of hours for high effective pressures at stick–slip asperities, and up to a several days at low effective pressures typical for inefficient drainage regions between asperities^49^. The absence of stick–slip episodes rupturing several asperities at once implies that stress transfer between asperities mainly happens via viscous creep through rate-strengthening bed regions but does not exclude elastic stress transfer between weakly mechanically coupled asperities.

Similar to repeating earthquakes at the Parkfield section of the San Andreas Fault^51^, stick–slip asperities at Rhonegletscher must not exceed a critical density and are only weakly mechanically coupled^52^. Otherwise, global ruptures of the self-organizing stick–slip failures eventually result from mutual triggering of asperities that are close to a state of failure similar as for earthquakes and no characteristic recurrence time of slips exist^53^. Moments, spatial and temporal distributions then scale as power laws^54^. For stick–slip asperities at Rhonegletscher, the lack of strong mechanical coupling between the asperities prohibits these large-scale failures resulting in moment dependent recurrence times revealing a memory of past events and a sub-critical state of self-organization^55^, whereas for a super-critical asperity density, randomness in recurrence times is expected^52^. On the other hand, critical asperity density could be a failure criterion for catastrophic glacial collapses^7^. Accordingly, we interpret regular glacial surge behavior as a sub-critical re-organization of the stress system similar to our sub-seasonal glacier velocity variations at Rhonegletscher.

Sliding seismicity at the glacier bed is strongly guided by the subglacial hydraulic system. Our findings show that concepts developed for earthquake faults apply to frictional processes at the glacier bed and may play an important role in the stability of small mountain glaciers and big ice streams. This also implies that glaciers offer a natural laboratory to study earthquake cycles and fault microseismicity subject to much higher variability in loading velocity and effective normal stress compared to tectonic strike-slip faults themselves. Relatively cheap in-situ glacial drilling experiments analogous to the San Andreas Fault Observatory at Depth, could image microseismic sliding at an unrivaled resolution.

## Methods

### Data acquisition

From September 28th 2017 to August 13th 2019, we operated three Lennartz 3D/BHs seismometers in an equilateral triangle with ~ 200 m side lengths in shallow (2–4 m) postholes in the ablation zone of Rhonegletscher (station RA51-53 of network 4D). The postholes had to be re-drilled several times during the deployment due to a surface melt of more than 10 m. We deployed these seismometers together with Nanometrics Taurus and Centaur digitizers sampling sensor output voltage at 500 Hz.

During the winter measurement period analyzed here (February 13th – March 20th, 2018), we extended the triangular array with four HL-6B 3-D geophones. We record their measurements with DIGOS DATA-CUBE^3^ digitizers sampling at 400 Hz. During the summer measurement period (July 21st – August 22nd, 2018), we extended the existing three-seismometer array with six additional Lennartz 3D/BHs sensors, each connected to a Nanometrics Centaur digitizer sampling at 500 Hz. Locations and specifications of all passive seismic stations are in the Supplementary Table S1.

Co-located with the three multiannual Lennartz 3D/BHs stations, we operated three PPM 2022-S13 GNSS sensors sampled at 15 s. Additionally, we installed a reference station on the orographic right moraine of the glacier for differential positioning in the data post-processing.

### Event catalog creation

#### Event detection

Signal discrimination between basal icequakes (stick–slip and tensile faulting) and surface icequakes mainly caused by crevassing, is based on high frequency content and short duration of the former. Elevated melt-induced seismic background noise in the ablation zone of alpine glaciers limits detectability of relatively weak stick–slip signals. In continuous spectrograms we automatically detect basal icequakes by searching for events with high (> 10 Hz) and broad frequency content. We verify these detections by the existence of distinct P- and S-wave arrivals and assess their frequency content and the relative power within both phases (Supplementary Fig. S1, S2).

#### Hierarchical clustering

Basal icequakes at glaciers are known to cluster temporally but also spatially resulting in virtually identical seismic waveforms^18,20^ (Supplementary Fig. S5). We use a hierarchical clustering to group events with similar waveforms. Orphans (events with low correlation with the rest of the catalogue) are discarded and event waveforms within clusters are stacked. These stacks are again clustered hierarchically to identify multiply detected clusters. The hierarchical clustering is designed to create clean rather than complete clusters, because the completeness will be achieved by subsequent template matching. For the further analysis we accept only clusters with mixed P-wave polarities. With this requirement we almost exclusively limit our study to the bed area directly below the seismometer arrays (i.e. one double-couple nodal plane of the seismic source must cross the array). On the other hand, this mixed polarity requirement ensures that we do not include basal crevassing events in our stick–slip catalogue.

#### Cluster cross-correlation

With the waveform stack of each stick–slip cluster, we run a normalized matched template cross-correlation search based on the algorithm EQcorrscan^56^ additionally accounting for template waveform SNR by only correlating station recordings with SNR above 3.5. By looking at the distribution of the cross-correlation coefficients, we define a threshold that separates events that we assume to be correct detections from false ones. This threshold is often clear from a kink in the cross-correlation coefficients’ histogram separating correctly matched events from other events or noise that are more frequently matched at low cross-correlation coefficients. (Supplementary Fig. S4).

#### Cluster location

For cluster location we use the probabilistic nonlinear hypocenter location algorithm NonLinLoc^30^ that accounts for picking and velocity model uncertainties. We use a P-wave velocity of $v_{p} = 3750\;{\text{ m/s}}$ and an S-wave velocity of $v_{s} = 1875 \;{\text{m/s}}$. Both values are based on active seismic measurements approximately 2 km down-glacier of our study site^57^. We include an uncertainty of 5% to these velocity values into our homogeneous velocity model, assuming that we only pick arrivals from direct waves that only travel through ice, but not through the underlying bedrock. The wave arrival time picking is done manually from waveform stacks that are output by the hierarchical clustering. Parameters for NonLinLoc are specified in Supplementary Table S5. Cluster locations are listed in Supplementary Table S2.

##### Seismic moment calculation

With knowledge of the source location, an estimate of shear fault mechanisms from direct P-wave polarities and typical elastic properties of glacier ice, we use the time integral of the horizontally polarized S-wave (SH) in the displacement seismogram to determine seismic moments of stick–slip events^58^:

5 $$ M_{0}  = \frac{{4{\pi \rho }_{i} {\upbeta }_{i}^{3} }}{{2F_{{SH}} }}{\textit{R}}\int\limits_{{t_{1} }}^{{t_{2} }} {u^{{SH}} } \left( t \right)dt $$

${\uprho }_{i } = 900 \;{\text{kg/m}}^{3}$ is the density of ice and ${\upbeta }_{i} = 1875 \;{\text{m/s}}$ is the S-wave velocity of ice. *F*_*SH*_ is the radiation pattern of a double couple source for a given focal mechanism^59^. The factor 2 accounts for the free surface amplification. The distance between hypocenter and recording seismic station is denoted by *R*. The integral over the ground displacement caused by the SH-wave *u*_*SH*_ is taken over the full SH-wave cycle from time *t*_*1*_ to *t*_*2*_. The ground displacement is obtained by integrating the recorded ground velocity seismogram and correcting for the instrument response.

Since some of our seismometers were installed in shallow boreholes, in a prior step the horizontal sensor orientation must be determined. This was done by horizontally rotating the seismogram of strong events from each asperity such that the P-wave amplitude is maximized on one horizontal component of the seismogram. From the known location of each station and each stick–slip cluster epicenter, the sensor orientation could be determined by calculating the mean rotation angle of all station-cluster pairs.

We manually pick the integration time window in Eq. (5) (*t*_*1*_ to *t*_*2*_) for the SH-wave of each cluster and use the alignment of the events in the stack to integrate for each event separately. For each event and station, we determine the seismic moment using Eq. (5) and searching for the orientation of the radiation pattern of a double-couple source with slip along the bed gradient that minimizes the standard deviation of seismic moments derived for each station. From the most suitable radiation patterns, we calculate the mean within cluster and thus obtain the fault orientation of each stick–slip asperity.

In order to account for the occasional borehole sensor re-installation in the seismic moment calculation, we use the amplitudes of the vertical components of the S-waves, which are always aligned with gravity, independent of the alignment of horizontal components. The vertical S-wave components scale linearly with integrated SH-waves and thus provide relative changes of seismic moments within each cluster. By scaling these relative changes with the calculated mean moment of the cluster from events outside of the redrilling period, we can precisely measure changes in intra-cluster seismic moments (Supplementary Fig. S7) and calculate absolute moments and their uncertainties. The resulting seismic moment catalog can be downloaded from the ETH Research Collection as stated in section Data availability.

### Recurrence time - seismic moment scaling

#### Power law fit

The recurrence time and seismic moment of repeating microseismic stick–slip events are expected to follow a power law relation $T_{r} \propto M_{0}^{1/6}$ for the case that aseismic creep exists^22^. We remove obvious outliers from the data by calculating the relative reduced $\chi^{2}$ change of a power law fit for each individual sample and remove data points that are not within two standard deviations of that sample distribution (Supplementary Fig. S8). We then apply a weighted linear fit to the remaining data points in double-logarithmic space ($log\left( {T_{r} } \right)$ vs. $log\left( {M_{0} } \right)$). The result is shown in Fig. 2a.

#### Spring-loaded slider-block model

We use a simple numerical 1D spring-loaded slider-block model on a frictional surface to model the recurrence time – seismic moment scaling. In this model, friction follows the Dietrich-Ruina law of rate-and-state friction^38,39^ expressed by:

6 $$ \tau = \left[ {\mu_{0} + a\;ln\left( {\frac{v}{{v_{0} }}} \right) + b\;ln\left( {\frac{{v_{0} \theta }}{L}} \right)} \right]\overline{\sigma } $$

where $\tau$ is shear stress and $\overline{\sigma }$ is the effective normal stress. The terms in brackets comprise the dynamic friction coefficient with slip velocity $v$*,* a reference velocity $v_{0}$ and the steady-state friction coefficient $\mu_{0}$ for $v = v_{0}$*.* The rate-strengthening parameter is *a*, the rate-weakening parameter is *b. L* Is the critical slip distance and $\theta$ is the state variable evolving according to:

7 $$ \frac{d\theta }{{dt}} = 1 - \frac{\theta v}{L} $$

We implement this law in a system of three differential equations. The first one is the regularized Dietrich–Ruina law solved for the slip velocity *v*,

8 $$ v = exp\left( {\frac{{ - \mu_{0} }}{a}} \right)v_{0} \cdot \left( {\frac{{v_{0} \theta }}{L}} \right)^{ - b/a} \cdot \left( {exp\left( {\frac{\tau }{{a \overline{\sigma }}}} \right) - 1} \right) $$

where we subtract 1 in the last term to start with 0 slip velocity. The second differential equation is the evolution of the state variable (Eq. 6) and the third differential equation expresses the temporal derivative of the shear stress ${\uptau }$ according to Hook’s law

9 $$ \dot{\tau } = k \left( {v_{load} - v} \right) $$

with the constant loading velocity $$v_{load} $$ of the spring in the slider-block model.

For $$\overline{\sigma } > \overline{\sigma }_{c}$$ with

10 $$ \overline{\sigma }_{c} = \frac{k L}{{ - \left( {a - b} \right)}} $$

being the critical effective normal stress, our rate-and-state friction law results in unstable sliding under quasi-static loading, producing stick–slip events^60^. We define the slip to be seismic, when the slip velocity reaches $v > 1\;{\text{mm/s}}$. All slip accommodated at lower velocities is assigned to aseismic creep.

Velocity weakening asperities at the glacier bed need to have a minimum size to slide seismically^37^, whereas we assume that their maximum size is limited by geometrical constraints of the glacier bed, like bedrock undulations. We thus assume that on average the asperity sizes and material properties are constant between the seasons, allowing us to use the most simplified case of a 1D spring-loaded slider-block model (Supplementary Fig. S9).

#### Bayesian inversion

We use an affine invariant Markov chain Monte Carlo Ensemble sampler^61^, to invert the recurrence time-seismic moment scaling of observed stick–slip events for parameters in the spring-loaded slider-block model subject to the rate-and-state friction law (Eqs. 8–10). These parameters include effective normal stress and loading velocity. We insert an ensemble of 3.8 million prior ensembles (48 walkers making 80,000 steps corresponding to 20 × the autocorrelation time) containing prior distributions of 11 parameters (Supplementary Table  S3), to the spring-slider model (Supplementary Fig. S10) and measure the likelihood to fit the observed recurrence time-seismic moment scaling by comparing the sample likelihood with the corresponding scatter point density from the observational data. Thus, we define the likelihood function for summer and winter data as the two-dimensional Kernel Density Estimation of the measurement distribution in the recurrence time-seismic moment phase space (Fig. 2a). Since we follow the assumption that neither material properties, rate-and-state friction parameters, nor the fault size change between winter and summer, we keep all input parameters (Supplementary Table S3) except for effective normal stress $\overline{\sigma }$ and loading velocity $v_{load}$ constant. This yields the variation in effective normal stress and loading velocity between summer and winter that is needed to reproduce the observed scaling. Figure 2b in the shows the modelled data and Fig. 2c shows the distribution of changes in loading velocity and effective normal stress between summer and winter. Figure S11 in the Supplementary Material shows a corner plot of the complete posterior parameter distributions and Table S4 lists the best fit values which are discussed in the Supplementary Notes.

### Time series processing

#### Seismic moment rate

We calculate the cumulative seismic moment that is released by all detected stick–slip asperities per time ($\mathop {M_{0} }\limits^{ \cdot }$) by summing up the seismic moments from individual events over six hours. Choosing this time interval, we cover sufficient events to produce a smooth activity measure of stick–slip asperities and are sensitive to multi-day variations in activity. We low pass filter resulting time series at a frequency of two days.

#### GPS velocity processing

From Global Positioning System (GPS) measurements on the glacier surface sampled at 15 s, velocities were calculated by using a static post processing method resulting in one velocity value per two hours. Outliers are removed by dropping data points of vertical velocity with a standard deviation larger than 3 m/d that result from adjustments of the antenna, and replacing them with linearly interpolated values. We also resample the data to the same resolution as the seismic moment rate and apply a Butterworth bandpass filter with corner frequencies of 3 days and 20 days.

#### Summer/winter GNSS velocity comparison

During the winter seismometer deployment, GNSS stations were buried by more than two meters of snow and therefore were not operational. This is why we cannot compare the winter stick–slip activity with ice flow velocity. However, winter GPS velocity measurements from November 2018 exist. Since already in November winter conditions are present at the measurement site, we assume that on monthly averages, these velocities are equal to the velocities that were present during the winter seismometer deployment. The distribution of measured velocities in Fig. 3b shows that winter surface velocities can be expected to be lower than summer surface velocities for most of the time. However, parts of the distribution overlap, reflecting summer night-time velocities and fast winter velocities.

## Supplementary Information

Supplementary Information.

## Data Availability

Seismometer data from stations RA51-RA63 of the 4D local glacier seismology network (https://doi.org/10.12686/sed/networks/4d) are archived at the Swiss Seismological Service and can be accessed via its web interface http://arclink.ethz.ch/webinterface/. All further data generated and analyzed during this study, like stick–slip icequake catalog, information about asperity source mechanisms, seismic station information, glacier surface GPS velocity and results from the Bayesian inversion are available in ETH Zurich’s Research Collection with https://doi.org/10.3929/ethz-b-000466250.
