# Supplementary material for: Changing friction at the base of an Alpine glacier
Source: Sci Rep. 2021 May 25;11:10872. doi: 10.1038/s41598-021-90176-9 (PMC8149391; doi:10.1038/s41598-021-90176-9)
Supplement: Supplementary file 1 — Supplementary Information. [file 41598_2021_90176_MOESM1_ESM.pdf]

Supplementary Information on

Changing Friction at the Base of an Alpine Glacier

by D. Gräff & F. Walter

Table of Contents

Supplementary Figures ... 2
- Stick-Slip Event Detection ... 2
- Hierarchical Clustering ... 2
- Matched Template Detection ... 3
- Stick-Slip Asperity Discrimination ... 4
- Seismic Moment Calculation ... 5
- Recurrence Time – Seismic Moment Scaling ... 6
- Spring-loaded Slider-Block Model ... 6
- Bayesian Inversion of Recurrence Time – Seismic Moment Scaling ... 7

Supplementary Tables ... 9
- Seismic Stations ... 9
- Stick-Slip Asperities ... 9
- Prior of Bayesian Inversion ... 10
- Best Fit Values of Bayesian Inversion ... 10
- NonLinLoc Input ... 10

Supplementary Notes ... 11
- Recurrence Time – Seismic Moment Scaling Velocity Normalization ... 11
- Interpretation of Posterior Inversion Parameter Values ... 11

Supplementary Bibliography ... 12

Supplementary Figures

Stick-Slip Event Detection

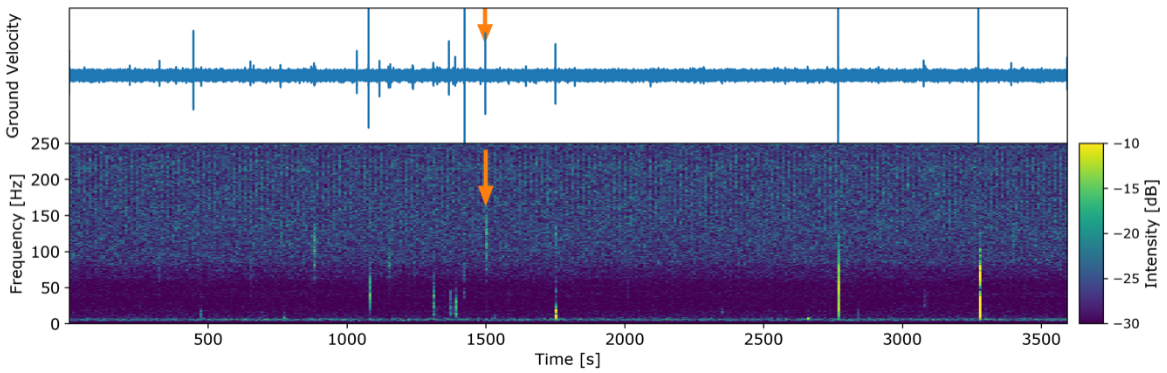

Figure S 1: Deep icequake detection. *Velocity seismogram and spectrogram of one-hour-long data from vertical component of station RA51. Orange arrows mark a stick-slip event.*

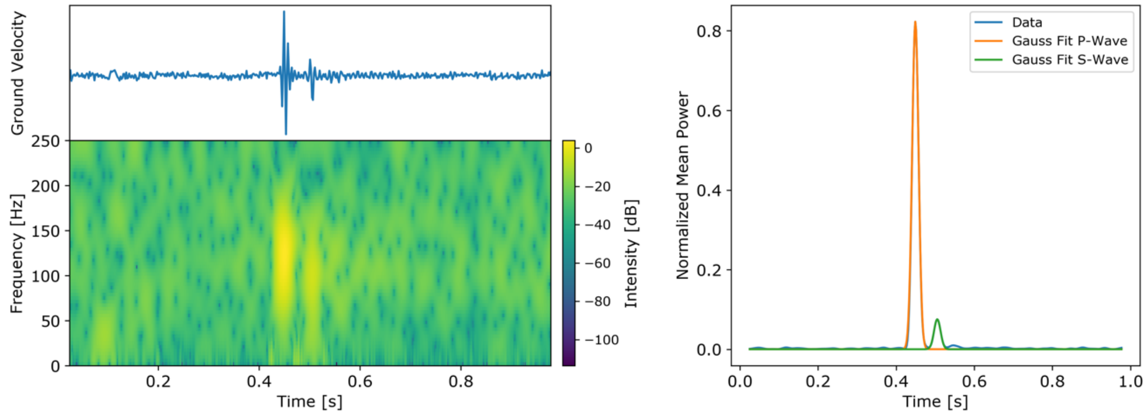

Figure S 2: Quality control of detected deep icequake*. The left panel shows a one-second-long zoom into the marked event from Fig. S3. Note the differing intensity color scale. The right panel shows the mean power of the P- and S-wave over all frequencies. Gaussians are fit to both waves.*

Hierarchical Clustering

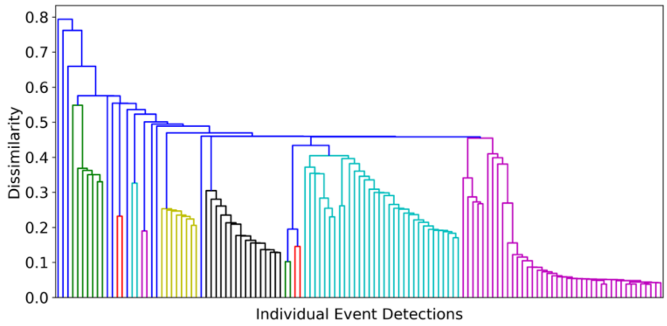

Figure S 3: Dendrogram of deep icequake detections. *Dissimilarity axis shows 1 minus the normalized correlation coefficient of connected detections. Clustering is carried out by applying inconsistency statistics on linkages.*

Matched Template Detection

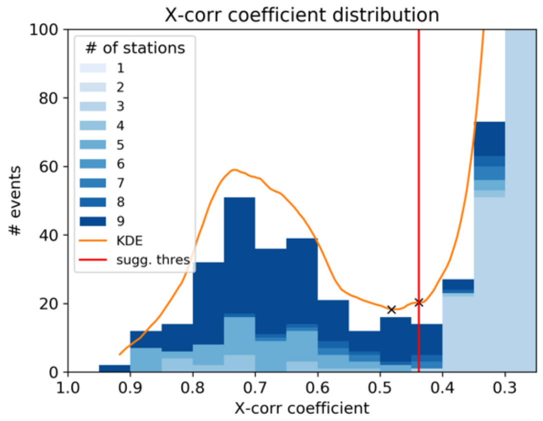

Figure S 4: Histogram and KDE of matched template detections. *Shown is the number of events detected with the matched template search for subsets of available stations with high SNR. Below a certain normalized correlation coefficients (here approximately 0.45) detection increases strongly, especially those for only a small set of available stations, because noise is increasingly detected.*

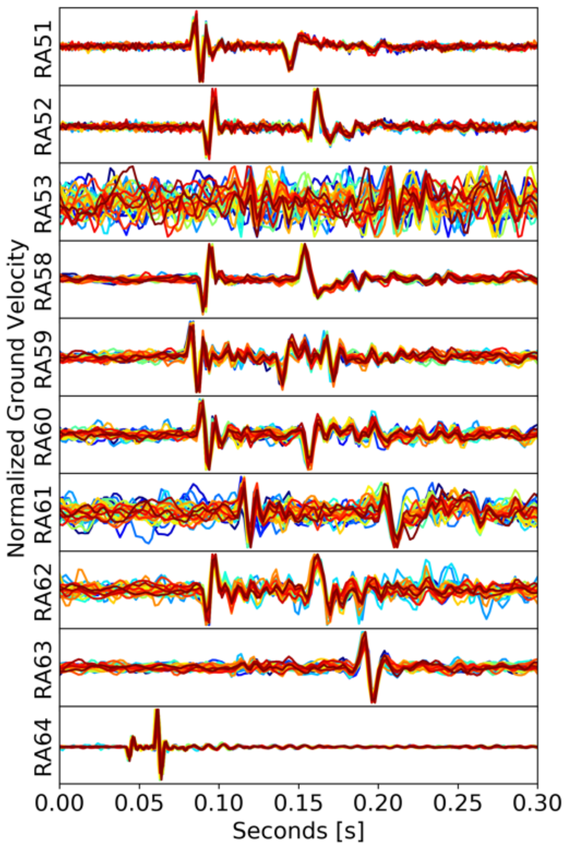

Figure S 5: Comparison of waveforms within a cluster. *Vertical seismograms of events within a stick-slip cluster for various seismic stations. All events within the same cluster arise from the same asperity. Each event is plotted in a different color of a rainbow colormap. In addition to the stations used for the data analysis in this paper, the record of a borehole seismometer RA64 close to the stick-slip asperity is shown. Note the mixed P-wave polarities.*

Stick-Slip Asperity Discrimination

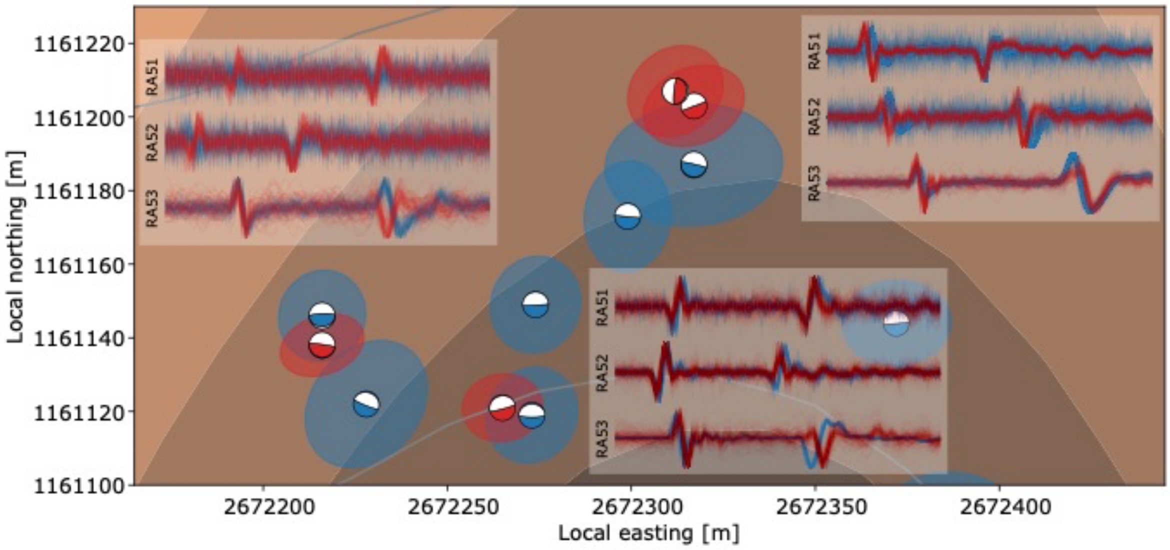

Figure S 6: Zoom into Fig1 a of the main text with waveforms*. Location of summer (red) and winter (blue) stick-slip asperities and their fault mechanisms indicated by beachballs. Red and blue shaded regions around beachballs show the horizontal projection of the 1σ uncertainty ellipsoid. Inserted waveforms show differences in P- and S-wave arrival for three summer-winter cluster pairs with overlapping location uncertainty at three seismic stations that were running for the winter and summer period. Waveforms belong to the closest red-blue cluster pair. Red lines belong to seismic signals from the summer asperity, blue lines to the winter asperity. Differences between waveforms of summer and winter clusters are larger than what would be expected from station displacement due to ice flow, indicating, that although location uncertainties overlap, their real locations differ.*

Seismic Moment Calculation

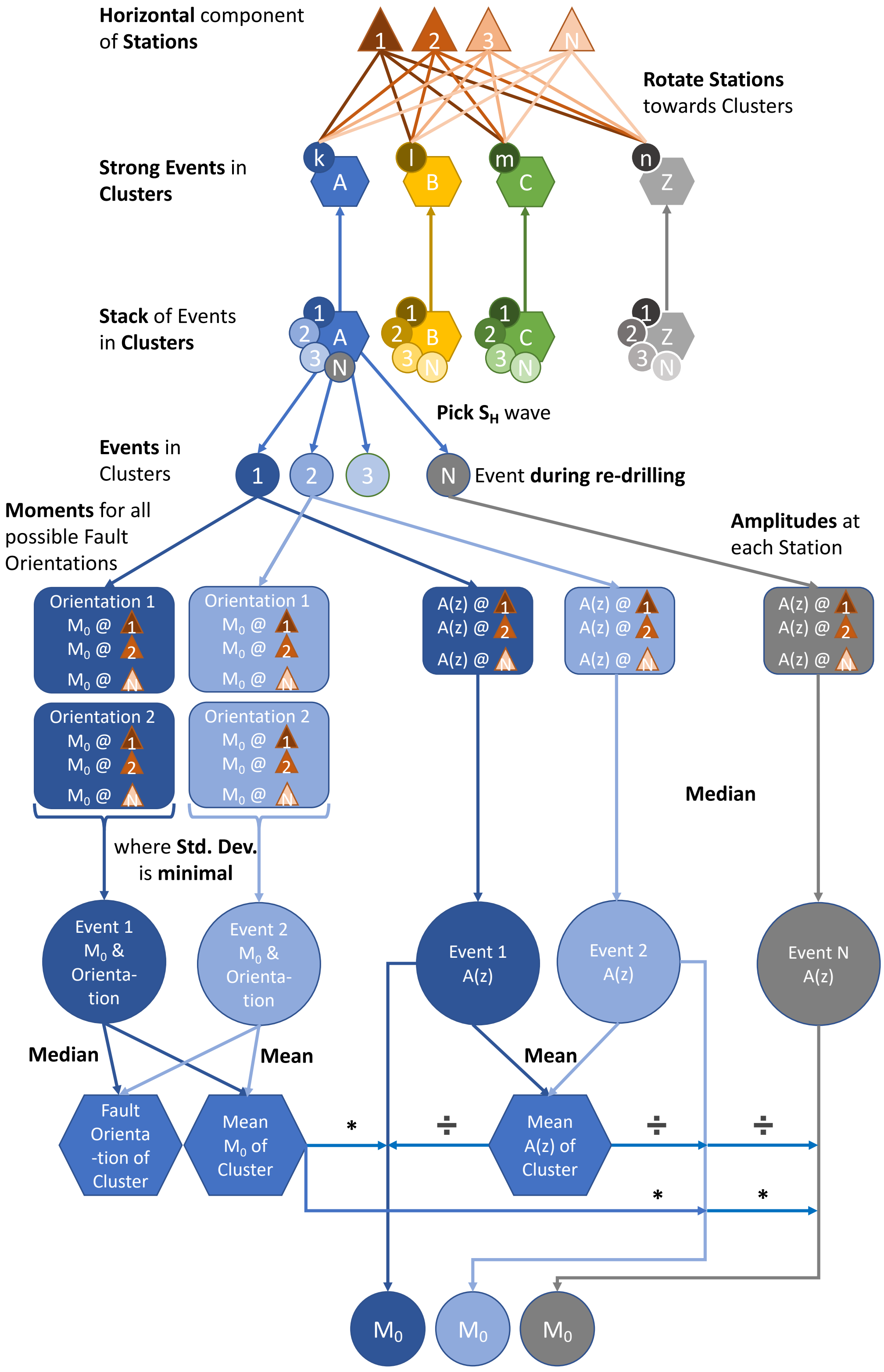

Figure S 7: ***Workflow for seismic moment calculation.*** *From top to bottom. Triangles represent seismic stations; circles correspond to stick-slip events and hexagons to stick-slip clusters. Blue shading represents individual stick-slip events that took place before or after the borehole sensor re-drilling period, and their corresponding workflow. Grey shading represents events that happened during the re-drilling phase. Re-drilling of sensors complicates the seismic moment (M_0) calculation substantially. Without re-drilling the workflow would only follow the left side of the scheme and terminate at the big blue shaded circles to the left.*

Recurrence Time – Seismic Moment Scaling

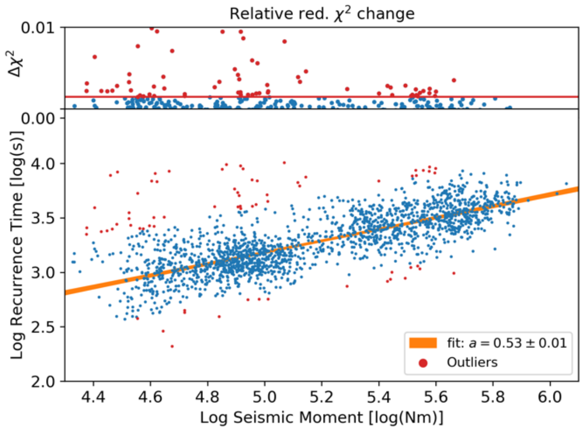

Figure S 8: Power law fit to recurrence time – seismic moment scaling. *In order to reach a better fit quality, outliers detected and removed with the leave-one-out method that iteratively leaves single measurements out from the fit and calculates the corresponding change in reduced χ^2. The detected outliers mostly arise from asperities terminating and re-starting their activity, resulting in long recurrence times.*

Spring-loaded Slider-Block Model

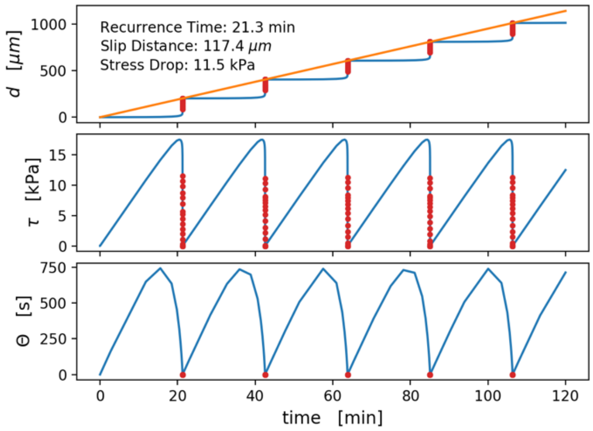

Figure S 9: Example output of spring-loaded slider block model. *We show in three panels the displacement of the slider block, the shear stress between the underlying surface and the slider block, and the state variable from the Dietrich-Ruina rate-and-state friction law. Marked with red dots are model steps during times when the sliding velocity reaches 1 mm/s, which we define as the threshold for seismic motion. The orange line in the first panel shows the spring loading.*

Bayesian Inversion of Recurrence Time – Seismic Moment Scaling

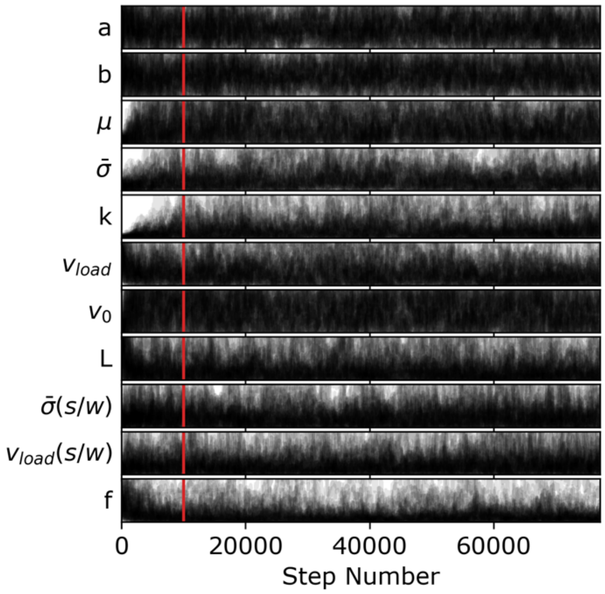

Figure S 10: Walker states of Monte Carlo Ensemble sampler. *For each input parameter of the Markov chain Monte Carlo Ensemble sampler, we run 48 walkers that are members of the ensemble. Their distribution is dependent on the positions of all other walkers. We discard samples from before step number 10,000, since we relate them to the burn-in phase, which can be seen clearest for parameters* $\mu, \bar{\sigma}$, *and* k.

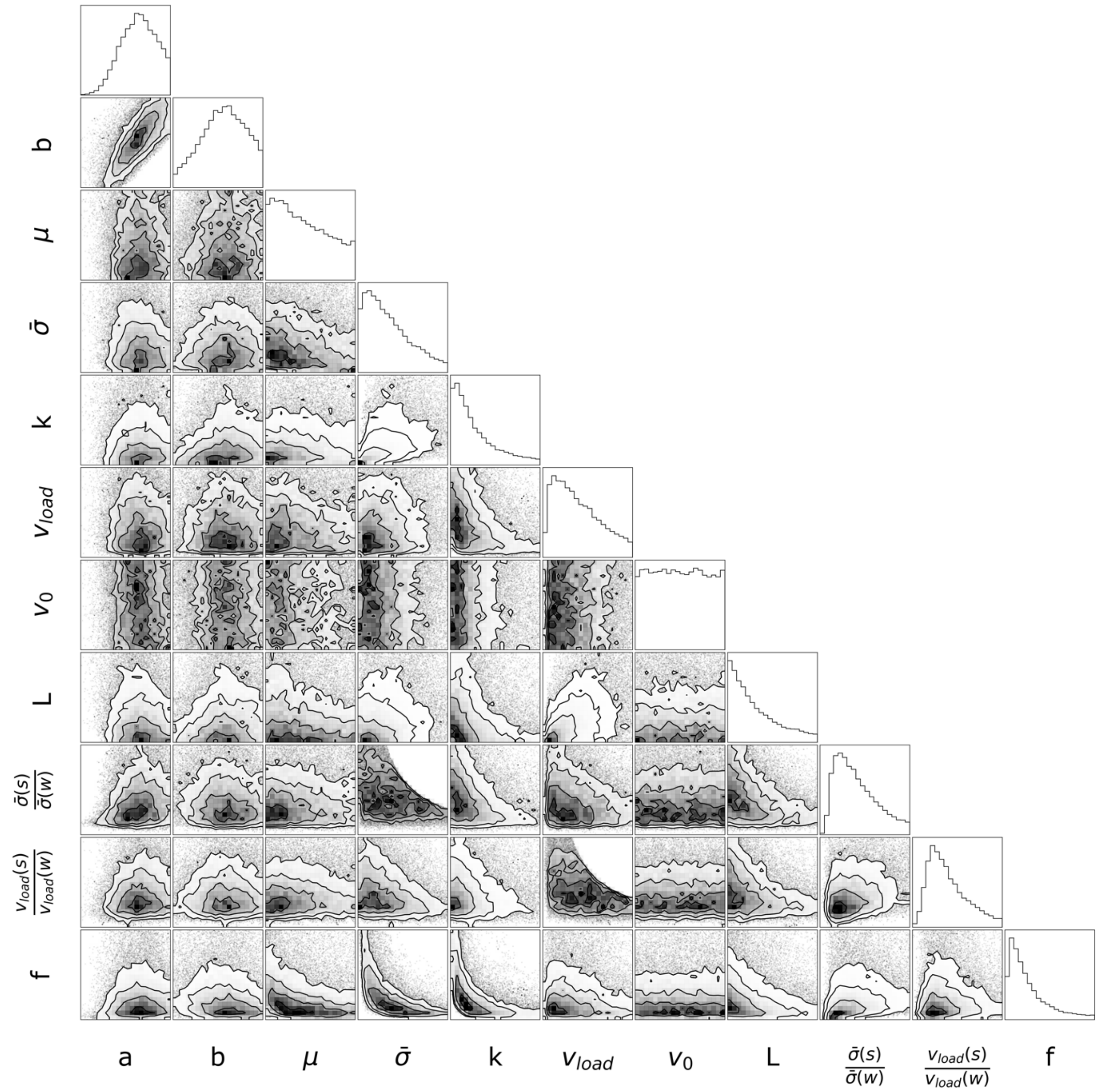

***Figure S 11: Corner plot of walkers from Monte Carlo Ensemble sampler**. For each combination of parameters of the Markov chain Monte Carlo Ensemble sampler, the mutual distributions of ensemble walkers are shown. Grey dots indicate the state of a single walker. Contours and grey shaded pixels indicate scatter point densities. Discarded walker samples are not included. Axis limits are at the 0th and 90th percentile of the posterior distributions. For a clearer visibility of the scatter point distribution, we only show 1% of almost 4 million model runs. Parameters are: **a**: rate-strengthening parameter, **b**: rate-weakening parameter, $\boldsymbol{\mu}$: steady-state friction coefficient at v=v_0, $\overline{\boldsymbol{\sigma}}$: effective normal stress, **k**: spring constant in spring-slider model, $\boldsymbol{v_{load}}$: loading velocity in spring-slider model, $\boldsymbol{v_0}$: sliding velocity of the steady state, **L**: critical slip distance, $\overline{\boldsymbol{\sigma}}(\boldsymbol{s})/\overline{\boldsymbol{\sigma}}(\boldsymbol{w})$: ratio of effective normal stress between summer and winter measurements, $\boldsymbol{v_{load}}(\boldsymbol{s})/\boldsymbol{v_{load}}(\boldsymbol{w})$: ratio of loading velocity in the spring-slider model between summer and winter measurements, **f**: factor linking the seismic stress drop to the seismic moment $\boldsymbol{M_0 = f \cdot \Delta\, \tau}$.*

Supplementary Tables

Seismic Stations

Station	Location LV95	Sensor Orientation	Sensor Model	Digitizer Model
**RA51**	2672400,1161165,2491	W: 247°, S1: 252°, S2: 192°	Lennartz 3D BH/s	Nanometrics Taurus
**RA52**	2672178,1161127,2490	W: 10°, S1: 26°, S2: 181°	Lennartz 3D BH/s	Nanometrics Taurus
**RA53**	2672314,1160959,2472	W: 357°, S1: 332°, S2: 108°	Lennartz 3D BH/s	Nanometrics Centaur
**RA54**	2672325,1161065,2480	0°	GFZ HL-6B 3-D	DIGOS DATA-CUBE^3
**RA55**	2672312,1161233,2494	0°	GFZ HL-6B 3-D	DIGOS DATA-CUBE^3
**RA56**	2672168,1161226,2503	0°	GFZ HL-6B 3-D	DIGOS DATA-CUBE^3
**RA57**	2672121,1161044,2483	0°	GFZ HL-6B 3-D	DIGOS DATA-CUBE^3
**RA58**	2672214,1161263,2500	S1: 67°, S2: 224°	Lennartz 3D BH/s	Nanometrics Centaur
**RA59**	2672341,1161264,2491	S1: 123°, S2: 10°	Lennartz 3D BH/s	Nanometrics Centaur
**RA60**	2672389,1161083,2476	S1: 101°, S2: 237°	Lennartz 3D BH/s	Nanometrics Centaur
**RA61**	2672426,1160955,2469	S1: 101°, S2: 195°	Lennartz 3D BH/s	Nanometrics Centaur
**RA62**	2672248,1161049,2471	S1: 98°, S2: 85°	Lennartz 3D BH/s	Nanometrics Centaur
**RA63**	2672281,1161407,2518	S1: 178°, S2 239°	Lennartz 3D BH/s	Nanometrics Centaur

Table S 1: Information about the seismic stations*. Locations given for RA51-RA57 were measured on March 22, 2018, and for RA58-RA63 on July 31, 2018 in Swiss LV95 coordinates. Ice flow is approximately 10 cm/d southward. Sensor orientation in degrees from north clockwise. W: indicates orientation for winter season, S1 and S2 for summer pre- and post-redrilling (2020-08-02, 2020-08-09).*

Stick-Slip Asperities

Asperity	Location LV95	Uncertainty Ellipsoid	Fault Orientation
**W01**	2672273, 1161119, 2296	22, 14, 16, -68°, 174°, 65 °	90±1, 14±1, -90
**W02**	2672274, 1161149, 2300	21, 14, 16, -68°, 153°, 71°	87±1, 3±1, -90
**W03**	2672228, 1161122, 2285	34, 17, 20, 63°, 26°, 306°	112±6, 10±3, -90
**W04**	2672372, 1161144, 2298	19, 14, 17, 34°, 86°, 4°	85±2, 3±2, -90
**W05**	2672221, 1161047, 2282	22, 14, 20, 57°, 36°, 130°	81±3, 5±2, -90
**W06**	2672299, 1161173, 2308	23, 14, 16, 54°, 3°, 147°	96±1, 2±1, -90
**W07**	2672168, 1160971, 2311	120, 18, 27, 81°, 20 °, 321°	52±6, 17±4, -90
**W08**	2672317, 1161187, 2301	30, 20, 25, 21°, 86°, 0°	283±0, 2±1, 90
**W09**	2672256, 1161021, 2282	25, 17, 20, 48°, 65°, 260°	101±3, 2±2, -90
**W10**	2672216, 1161146, 2313	20, 13, 15, -64°, 150°, 80°	268±2, 15±1, 90
**W11**	2672389, 1161091, 2283	26, 15, 18, -11°, 100°, 178°	78±4, 2±3, -90
**S01**	2672112, 1161255, 2348	48, 20, 27, 79°, 104°, 322°	96±8, 9±5, -90
**S02**	2672317, 1161203, 2311	32, 13, 17, -79°, 50°, 203°	69±2, 2±2, -90
**S03**	2672312, 1161207, 2298	23, 13, 17, -73°, 148°, 87°	186±3, 11±2, 90
**S04**	2672280, 1161262, 2318	22, 11, 18, 61°, 46°, 294°	74±1, 7±1, -90
**S05**	2672216, 1161138, 2307	16, 10, 14, -59°, 70°, 181°	278±4, 6±3, 90
**S06**	2672307, 1161341, 2329	21, 12, 17, -27°, 81°, 224°	99±3, 11±4, -90
**S07**	2672265, 1161121, 2293	16, 11, 14, -80°, 39°, 226°	75±2, 8±2, -90

Table S 2: Stick-slip asperity location and orientation. *Winter asperities starting with 'W', summer asperities with 'S'. Column 'Location' states the most probable hypocenter in Swiss LV95 coordinates. Uncertainty ellipsoid states the semi-major, semi-minor, semi-intermediate axis length, and major axis plunge, azimuth and rotation of the uncertainty rotation ellipsoid. The column 'Fault Orientation states strike, dip and rake of the fault. We assume slip along the bed gradient.*

Prior of Bayesian Inversion

Parameter	Prior	Unit	Reference
**a**	$e^{-((x-0.02)/0.01)^2}: a \in [0.01, 0.05], < b$	-	[1-3]
**b**	$e^{-((x-0.03)/0.01)^2}: b \in [0.02, 0.08], > a$	-	
**μ**	$1: \mu \in [0, 0.5]$	-	[1, 4]
**k**	$1: k \in [1 \cdot 10^3, 5 \cdot 10^9]$	N/m	[3, 5-10]
$\mathbf{v_0}$	$1: v_0 \in [10^{-9}, 10^{-5}]$	m/s	[11]
**L**	$1: L \in [10^{-7}, 10^{-5}]$	m	[11]
**f**	$1: f \in [0, 100]$	-	free fitting parameter
$\bar{\boldsymbol{\sigma}}$	$1: \bar{\sigma} \in [0, 18 \cdot 10^5]$, $< k \cdot L/(-(a-b))$	Pa	[12, 13]
$\mathbf{v_{load}}$	$1: v_{load} \in [1.2 \cdot 10^{-9}, 2.3 \cdot 10^{-7}], < v_0$	m/s	[14]
$\bar{\boldsymbol{\sigma}}(\boldsymbol{s/w})$	$1: \bar{\sigma}(w) \cdot \bar{\sigma}(s/w) \in [0, 18 \cdot 10^5]$, $< k \cdot L/(-(a-b))$	-	free fitting parameter
$\boldsymbol{v_{load}(s/w)}$	$1: v_{load}(w) \cdot v_{load}(s/w) \in [1.2 \cdot 10^{-9}, 2.3 \cdot 10^{-7}], < v_0$	-	free fitting parameter

Table S 3: Prior of Bayesian inversion input. *All parameters besides a and b have constant probabilities within the given interval and have 0 probability elsewhere. The prior of a and b was chosen as a Gaussian distribution, because model runs favored values at the upper end of their given prior interval (we choose to stay in consensus with the given references).*

Best Fit Values of Bayesian Inversion

Parameter	16th percentile	50th percentile	84th percentile	Unit
**a**	0.019	0.023	0.027	-
**b**	0.026	0.031	0.036	-
**μ**	0.05	0.18	0.37	-
**k**	0.2	0.6	1.8	GN/m
$\mathbf{v_0}$	---	---	---	m/s
**L**	0.5	1.6	4.6	μm
**f**	0.9	2.3	7.1	-
$\bar{\boldsymbol{\sigma}}$	0.07	0.26	0.61	MPa
$\mathbf{v_{load}}$	2.1	5.6	12.0	mm/d
$\bar{\boldsymbol{\sigma}}(\boldsymbol{s/w})$	1.6	3.0	5.9	-
$\boldsymbol{v_{load}(s/w)}$	0.9	1.6	3.3	-

Table S 4: Best fit values of Bayesian inversion. *16 ,50, and 84% quantiles of posterior distributions are given. We expect an uncertainty of ~5% on all values due to the termination criterium of the inversion. The posterior distribution of v_0 is flat and therefore no fitting values are given.*

NonLinLoc Input

Setting	Parameter	Value
**Location grid**	Cell number X/Y/Z	200/300/100
	Initial cell size X/Y/Z	5/5/5 m
	Highest cell elevation	2600 m
	Grid projection type	Lambert Conformal Conic projection
	Center of grid	WGS-84: 46.592 8.375
	P-wave velocity	3,750 m/s (3,560 to 3,940 m/s)
	S-wave velocity	1,875 m/s (1,780 to 1,970 m/s)
**Location algorithm**	Location search type	Octree
	Location method	EDT_OT_WT*
	Typical travel time uncertainty	0.002 s
	Fraction of travel time uncertainty	0.05 → 5% velocity model uncertainty
	Max travel time error	0.003 s

Table S 5: NonLinLoc input parameters. *P- and S-wave velocities derived from active borehole measurements at the lower tongue of Rhonegletscher. *EDT_OT_WT calculates Equal Differential Time (EDT) likelihood function and weights the EDT-sum probabilities by the variance of origin-time estimates over all pairs of readings. For further description of parameters, see [15].*

Supplementary Notes

Recurrence Time – Seismic Moment Scaling Velocity Normalization

We derive a theoretical relation for the event recurrence time and the seismic moment from the standard definition of seismic moment [16]

$$M_0 = \gamma \pi r^2 \Delta d_{seis} \qquad \text{(S1)}$$

where γ is the shear modulus, Δd_{seis} is the seismic slip, and r is the radius of a circular rupture. Combined with the static stress drop [17, 18]

$$\Delta\tau_s = \frac{7\pi\gamma\Delta d_{seis}}{16r} \qquad \text{(S2)}$$

and assuming all slip is released seismically $\Delta d_{total} = \Delta d_{seis} = T_r v_l$ this results in a scaling [19]:

$$T_r = \frac{\Delta\tau_s^{2/3} M_0^{1/3}}{1.81\gamma v_l} \qquad \text{(S3)}$$

For the case of purely seismic slip, we expect that the event recurrence time is proportional to the inverse of the loading velocity v_l. Thus, if we assume $\Delta\tau_s = const.$ similar to [20], we can normalize our summer recurrence time measurements by summer loading velocities

$$\mathrm{T_r(norm)} = \frac{\mathrm{v_{load}(summer)}}{\mathrm{v_{load}(winter)}} \cdot \mathrm{T_r(summer)} \qquad \text{(S4)}$$

However, for $v_{load}(summer) > v_{load}(winter)$ (as measured), this only leads to even larger recurrence times in summer when accounting for changes in loading velocity. Smaller summer event recurrence times would be needed though, to adapt our measurements from a scaling of $T_r \propto M_0^{0.53\pm0.01}$ to a lower power of $T_r \propto M_0^{1/6}$ as observed for microseismic tectonic strike-slip faults [20].

Interpretation of Posterior Inversion Parameter Values

Best fitting values of the inversion parameters from Table S4 give indications about subglacial conditions at basal stick-slip asperities:

- $\bar{\boldsymbol{\sigma}}$: Approximately 10-15% of the ice overburden pressure for the winter season indicating conditions close to flotation. Approximately threefold value in summer, similar to borehole water pressure measurements.
- $\mathbf{v_{load}}$: Realistic sliding velocity (~5% of surface velocity) that is in accordance with direct measurements of sliding velocity further down-glacier [21].
- $\boldsymbol{\mu}$: Friction coefficient posterior only weakly constrained, but higher than for pure ice at the pressure melting point on hard bedrock and till. The friction coefficient posterior is lower than for rock-on-rock sliding, but comparable to debris laden ice on till, which points towards the existence of a frozen fringe over a soft bed at stick-slip asperities [22, 23].
- **k:** Spring constant in the 1D spring-slider model corresponds to the shear modulus in 3D. Posterior values range between shear moduli expected for subglacial till in the MPa range [5, 6], and ice in the GPa range [10].

- **a-b:** The low value of 0.008 indicates only mild rate-weakening as expected for ice at the pressure melting point [24].
- **L:** Values in the micron range point towards subglacial till with a high clay content [25].

Best fit posterior inversion values do not allow for a strict interpretation of material properties of the sliding interfaces. The stiff spring constant is pointing towards hard bedrock conditions, whereas low friction and characteristic slip distance point towards a soft bed. Borehole camera observations at a stick-slip asperity show fine grained till which reacts on changes in subglacial water pressure, and ice with rock intrusions up to a few meters above the ice-till interface, pointing towards spatially limited soft bed conditions at the asperities [26]. The bedrock of Rhonegletscher can be assessed from the glacier forefield, where the glacier recently retreated. Undulations of bedrock filled with till between humps confirm the existence of confined till patches at the glacier bed [27].

Supplementary Bibliography

1 McCarthy, C., H. Savage & M. Nettles. Temperature dependence of ice-on-rock friction at realistic glacier conditions. *Philosophical Transactions of the Royal Society a-Mathematical Physical and Engineering Sciences* **375**, 20150348, (2017).

2 Lipovsky, B. P. & E. M. Dunham. Slow-slip events on the Whillans Ice Plain, Antarctica, described using rate-and-state friction as an ice stream sliding law. *Journal of Geophysical Research: Earth Surface* **122**, 973-1003, (2017).

3 Lipovsky, B. P. *et al.* Glacier sliding, seismicity and sediment entrainment. *Annals of Glaciology* **60**, 182-192, (2019).

4 Zoet, L. K. & N. R. Iverson. A healing mechanism for stick-slip of glaciers. *Geology* **46**, 807-810, (2018).

5 Iverson, N. R. Shear resistance and continuity of subglacial till: hydrology rules. *Journal of Glaciology* **56**, 1104-1114, (2010).

6 Iverson, N. R. Coupling between a glacier and a soft bed: II. Model results. *Journal of Glaciology* **45**, 41-53, (1999).

7 Fischer, U. H. & G. K. C. Clarke. Stick-slip sliding behaviour at the base of a glacier. *Annals of Glaciology, Vol 24, 1997* **24**, 390-396, (1997).

8 Helmstetter, A., B. Nicolas, P. Comon & M. Gay. Basal icequakes recorded beneath an Alpine glacier (Glacier d'Argentière, Mont Blanc, France): Evidence for stick-slip motion? *Journal of Geophysical Research: Earth Surface* **120**, 379-401, (2015).

9 Hudson, T. S. *et al.* Icequake source mechanisms for studying glacial sliding. *Journal of Geophysical Research: Earth Surface* **125**, e2020JF005627, (2020).

10 Hobbs, P. V. Ice physics. (Oxford university press, 2010).

11 Scholz, C. H. Earthquakes and friction laws. *Nature* **391**, 37-42, (1998).

12 Gräff, D., F. Walter & B. P. Lipovsky. Crack wave resonances within the basal water layer. *Annals of Glaciology*, 1-9, (2019).

13 Rutishauser, A., H. Maurer & A. Bauder. Helicopter-borne ground-penetrating radar investigations on temperate alpine glaciers: A comparison of different systems and their abilities for bedrock mappingHelicopter GPR on temperate glaciers. *Geophysics* **81**, WA119-WA129, (2016).

14 GLAMOS. The Swiss Glaciers 2015/16 and 2016/17. Vol. 137/138 (Cryospheric Commission (EKK) of the Swiss Academy of Sciences (SCNAT), 2018).

15 Lomax, A., J. Virieux, P. Volant & C. Berge-Thierry. in *Advances in seismic event location* 101-134 (Springer, 2000).
16 Aki, K. & P. G. Richards. Quantitative seismology. (2002).
17 Eshelby, J. D. The determination of the elastic field of an ellipsoidal inclusion, and related problems. *Proceedings of the royal society of London. Series A. Mathematical and physical sciences* **241**, 376-396, (1957).
18 Keylis-Borok, V. On estimation of the displacement in an earthquake source and of source dimensions. *Annals of Geophysics* **53**, 17-20, (2010).
19 Beeler, N. M., D. L. Lockner & S. H. Hickman. A simple stick-slip and creep-slip model for repeating earthquakes and its implication for microearthquakes at Parkfield. *Bulletin of the Seismological Society of America* **91**, 1797-1804, (2001).
20 Chen, K. H., R. M. Nadeau & R. J. Rau. Towards a universal rule on the recurrence interval scaling of repeating earthquakes? *Geophysical Research Letters* **34**, (2007).
21 Gräff, D., F. Walter & A. Bauder, *Borehole Measurements and Basal Velocity of Rhonegletscher. Analysis of Borehole Measurements and Determination of the Basal Sliding Velocity of Rhonegletscher*, in *15th Swiss Geoscience Meeting (SGM 2017), Davos, Switzerland, November 17-18, 2017*. 2017, ETH Zurich, Laboratory for Hydraulics, Hydrology and Glaciology.
22 Thompson, A. C., N. R. Iverson & L. K. Zoet. Controls on Subglacial Rock Friction: Experiments With Debris in Temperate Ice. *Journal of Geophysical Research: Earth Surface* **125**, e2020JF005718, (2020).
23 Zoet, L. K. *et al.* Application of Constitutive Friction Laws to Glacier Seismicity. *Geophysical Research Letters* **47**, e2020GL088964, (2020).
24 Zoet, L. K. *et al.* The effects of entrained debris on the basal sliding stability of a glacier. *Journal of Geophysical Research-Earth Surface* **118**, 656-666, (2013).
25 Lipovsky, B. P. & E. M. Dunham. Tremor during ice-stream stick slip. *Cryosphere* **10**, 385-399, (2016).
26 Gräff, D. & F. Walter. Videos of Subglacial Till Dynamics. (2019).
27 Walter, F. *et al.* Distributed acoustic sensing of microseismic sources and wave propagation in glaciated terrain. *Nature Communications* **11**, 2436, (2020).
